# Tympanitis

**Published:** 1880-10

**Authors:** Geo. H. Berns


### ART. XXI.—TYMPANITIS.

LECTURE ON EQUINE PRACTICE, COLUMBIA VETERINARY COLLEGE.

BY GEO. H. BERNS, D. V. S.

THE second form of colic is known as tympanitis, flatulent, or wind colic.

This form differs from the last in every particular, in its pathology, symptoms, and treatment ; therefore, the practitioner of veterinary medicine cannot be too careful in drawing a correct line of distinction between these two important disorders. While in spasmodic colic an anti-spasmodic drench will in most all uncomplicated cases speedily relieve the suffering horse, the same remedies would in flatulent colic not only be useless as therapeutic agents, but most of them would have a tendency to aggravate the trouble.

In tympanitis (from tympanum, a drum) there is a distended condition of the abdomen. The stomach or intestines, or both, become distended with gases, generated in these organs from

* Beitr. zur Biol. d. Pflanzen, Band i., Heft 2, p. 146. It is allowed by Cohn that this classification is only provisional, though the orders are natural. Beit. zur Biol. d. Pflanzen, Band. ii., p, 274.

food, which, instead of being properly acted upon by the gastric and intestinal secretions, digested and assimilated, undergoes the process of fermentation, gases being generated, and tympanitis is the result.

Among the many causes which are productive of this form of trouble, improper and injudicious feeding again heads the list. Too large a quantity of food of any description, if hastily swallowed by an animal fatigued by a long journey, is very apt to bring on intestinal disorders, whilst dry food, such as oats or corn, is productive of spasmotic colic; soft or green food is more likely to cause the form of colic we are now considering.

Cooked food of any kind, mashes, grass, clover, or vegetables, will readily undergo fermentation in the stomach of a horse, if they have been given in too large a quantity or at improper times. Horses which are kept in city stables and fed almost exclusively on oats and hay, will, if they get an opportunity, eagerly devour inordinate quantities of these articles of food, and if proper care is not exercised by the attendants in charge, many cases of flatulent colic are sure to occur.

Again, in horses suffering from acidity of the stomach, and weak and impaired digestive powers, flatulent colic frequently occurs without any cognizable extrinsic cause. It sometimes comes on during the progress of another disease, and if there be no extrinsic cause to produce it, it indicates that the digestive powers of the animal are so exhausted as to be bordering upon dissolution. Tympanitis sometimes occurs during an attack of spasmodic colic, and when it does, it proves to be a most serious complication. It may also be produced by mechanical obstructions of the intestinal canal, such as tumors, calculi, etc.

*Symptoms*—The expression of pain, though not so acute, is more constant than in spasmodic colic. The horse will paw, look to his flank, and make frequent attempts to lie down; when down, he will roll, or he may remain quiet for some considerable time. The act of lying down or rolling is carefully performed. A horse may stand in a corner of his stall for hours with his head depressed, an anxious and painful expression of countenance, and now and then turn his head and look dolefully at his flank, or elevate his head and retract his upper lip. There is always more or less distension of the abdomen, but if the collection of gases is chiefly confined to the large intestines, the abdomen frequently becomes of an enormous size, the walls are tense, and produce a resonant sound on percussion. The respirations are labored, increased in frequency, and thoracic in all cases of flatulency; but if the collection of gases is principally confined to the stomach, this symptom is greatly aggravated with eructations of gases and attempts at vomiting, while the distention of the abdomen may be hardly noticeable. The circulation is very much disturbed, the pulse increased in frequency, and soon becomes feeble. The legs, ears, and face become cold, cold sweats bedew the body, the animal reels to and fro, becomes more or less delirious, and if not relieved, death ensues either from asphyxia, due to pressure from the abdominal viscera against the diaphragm; blood poisoning from absorption of deliterious gases, or from rupture of the stomach or some portion of the intestines from over-distention, or from general exhaustion.

If a horse really succeeds in vomiting up solids or fluids, it is generally held that a rupture of the muscular coat of the stomach has taken place. This as a rule is, no doubt, correct; but there are numerous instances on record, and I recall one case, which came under my observation, where the horse vomited freely, and yet made a good recovery.

*Treatment*—A cathartic is advisable in all cases, followed by stimulants and agents which have a tendency to absorb or neutralize the gases. From 3 to 5 dr. of the compound extract of colocynth., ½ oz. of tincture of opium, ½ oz. of aromatic spts. of ammonia, given in 12 oz. of linseed oil, constitute probably as good a drench as can be administered. This should be followed by 1 oz. doses of the aromatic spts. of ammonia every hour. The spts. of ammonia must be well diluted to prevent irritation of the mouth and fauces. Charcoal, chloride of lime, turpentine, and many other agents are recommended by writers on veterinary medicine, and may be tried; but I consider the aromatic spts. of ammonia as efficient as any of them. Enemas of warm water and soap, and gentle exercise aid the action of the cathartic and assist in the expulsion of gases. Unfortunately these remedies sometimes prove too slow in their action, and many horses are lost on this account; therefore a surgical operation is resorted to, by which the accumulated gases are expelled from the viscera by mechanical means. It consists in puncturing into the coecum or colon with a small trocar, and is known as "*Paracentesis abdominis.*" It is by no means a new operation, for *Riem*, *Burgelat*, *Chabert*, and others performed it almost a hundred years ago.

German and French veterinarians speak of it in very favorable terms, whilst English authors do not seem to think much of it.

For the past few years it has been practiced to a considerable extent in this country, and if all the reports can be credited, the bulk of the evidence is certainly very much in its favor. A veterinarian reports in the *American Veterinary Review* some eight or ten cases, and if my memory serves me, I think he claims that all of them recovered. I have not been as successful as this gentleman has, for I punctured in two cases which resulted fatally, both horses dying; not from the effects of the operation, however, for in both instances death ensued within an hour after operating, and the post mortem revealed that the punctures in the walls of the intestines had closed up so effectually, that it required a very close examination on my part to find the places; there were no traces of peritonitis. The probabilities are that the operations were delayed too long, and that both horses died from exhaustion or blood poisoning. I think this operation advisable in cases where there is a large accumulation of gases in the coecum or colon, and where enemas, gentle exercise, and the recommended remedies do not seem to afford any relief in two or three hours. Too much time should not be lost; for, in my opinion, the dangers of death from the accumulated gases are far greater than those that may result from the operation.

The serious consequences that may follow the operation are peritonitis from the admission of air or fæcal matters into the peritoneal cavity, or from accidently puncturing a small blood-vessel.

The operation is a very simple one, and the only instruments required are a small trocar and a sharp-pointed bistoury.

The right flank from four to five inches in front of the anterior angle of the ilium, is designated by the most authorities as the best place to puncture, and no doubt it is in the majority of cases; but when we consider how very loosely the horse's intestines are attached, and that they are constantly changing their relative positions, which is particularly the case in horses suffering from colic, it is impossible to say with any degree of certainty with what portion of the gut our trocar will come in contact. I think it more practicable to puncture that portion of the abdomen where the largest collection of gases can be detected, for the danger of peritonitis is as great in one region as it would be in another.

A very small incision should be made through the skin with a small bistoury, the skin then drawn to one side, and the trocar plunged rapidly into the distented gut. The object of cutting through the skin first and then drawing it to one side before the trocar is introduced, is to prevent the opening in the skin from coming in direct opposition to the opening through the abdominal walls, peritoneum and coats of the intestines, and by this means prevent the admission of air. As soon as the stylet is withdrawn, large quantities of gases, chiefly composed of sulphuretted hydrogen, will escape. Should the canula become clogged up with fæcal matters, the stylet must be re-introduced and the obstruction removed. If but little gas escapes, the chances are, that some portion of the small intestines have been punctured, and a second puncture may be made through the same opening in the skin in another direction, or a new place may be tried. It is advisable that the stylet be re-introduced before the trocar is withdrawn, for the purpose of removing any fæcal matters that may be clinging to the internal part of the tube, and accidently drop into the peritoneal cavity on withdrawing the instrument.

*Borgniez* injected 2 oz. of the tincture of aloes through the tube and reported good results.

*Stockfleth* tried the same experiment, and reported death from peritonitis on the ninth day after the operation.

I think I would content myself with the administration of cathartics per mouth, rather than run the risk of setting a violent irritation by the injection of cathartic remedies through the tube.

After most of the gases have escaped, and the instrument has been withdrawn, the condition of the animal should be carefully noticed, and if the pulse continues feeble, legs and ears colder than natural, the stimulating plan of treatment must be kept up. Alcoholic stimulants may then take the place of the ammoniacal friction, stimulating embrocations to the extremities, and flannel bandages will aid in equalizing the disturbed circulation. The after-treatment must be conducted on general principles.

